# Complications of tunneled central venous catheter placement: a narrative review of risks, prevention, and management strategies

**DOI:** 10.3389/fradi.2025.1684246

**Published:** 2025-11-20

**Authors:** Fabio Corvino, Felice D'Antuono, Francesco Giurazza, Claudio Carrubba, Alessandro Punzi, Antonio Corvino, Massimo Galia, Raffaella Niola

**Affiliations:** 1 Section of Radiology, Department of Biomedicine, Neuroscience and Advanced Diagnostics (BiND), University Hospital “Paolo Giaccone”, Palermo, Italy; 2 Interventional Radiology Department, AORN “A. Cardarelli”, Naples, Italy; 3 Medical, Movement and Wellbeing Sciences Department, University of Naples “Parthenope”, Naples, Italy

**Keywords:** hemodialysis, tunneled cuffed catheter, tunneled central venous catheter, vascular access complications, infection, thrombosis, central venous stenosis, catheter dysfunction

## Abstract

**Background:**

Tunneled cuffed catheter (TCC) remains a crucial vascular access option for patients undergoing hemodialysis, particularly in those who are not candidates for arteriovenous fistulas or grafts. However, placement carries immediate and delayed complications.

**Objective:**

This narrative review aims to provide a comprehensive overview of the complications encountered during and after the placement of a TCC for hemodialysis, highlighting current evidence, risk factors, prevention strategies, and management approaches.

**Methods:**

A critical selection of relevant literature was performed through PubMed and Scopus databases, focusing on articles published in the last two decades. Particular attention was given to studies reporting on mechanical, infectious, thrombotic, and late-onset complications, as well as technical factors influencing outcomes.

**Results:**

Complications of TCCs can be classified as immediate (e.g., arterial puncture, pneumothorax, bleeding), early (e.g., catheter malposition, exit-site infections), and late (e.g., central venous stenosis, catheter-related bloodstream infections, thrombosis). Patient- and procedure-related factors increase risk. Ultrasound and fluoroscopy, strict sterility, and timely management reduce complications rates.

**Conclusion:**

TCCs are indispensable in selected patients, but understanding their complications is key to patient safety and outcomes. Optimal outcomes depend on accurate patient selection, operator expertise, and standardized post-placement care.

## 1 Introduction

Hemodialysis is a life-sustaining therapy for patients with end-stage renal disease, and its effectiveness depends on reliable vascular access. While AVFs and AVGs are the preferred long-term solutions, and PD represents a valuable alternative in selected patients, a significant proportion of individuals either cannot undergo these procedures or require immediate access before maturation. In these cases, TCCs provide immediate accesso to the central venous system (1).

Despite their clinical utility, TCCs are associated with a wide range of complications that may compromise patient outcomes and healthcare resources. Complications may occur at insertion, soon after placement, or during long-term use, and include mechanical problems, infection, thrombosis, and late vascular damage. Safe insertion requires careful technique, imaging guidance, and awareness of patient anatomy and comorbidities (2, 3).

Despite available guidelines and protocols, complication rates remain high, especially in patients with multiple risk factors or prior catheter-related issues. Variations in practice patterns and continuous evolution of catheter design also limit standardization (4, 5).

This narrative review summarizes the main complications of TCC for hemodialysis, outlining their mechanism, risk factors, prevention, and management.

### 1.1 Literature search and methodology

The narrative review was informed by a structured search of PubMed/MEDLINE, Embase, and Scopus covering the period from January 2000 to March 2025. The search strategy combined terms such as hemodialysis, tunneled cuffed catheter, tunneled central venous catheter, vascular access complications, infection, thrombosis, central venous stenosis, catheter dysfunction, and catheter management. Additional studies were identified by screening the reference lists of retrieved articles and relevant guideline documents. We included original research articles, systematic reviews, meta-analyses, and clinical guidelines published in English, focusing on adult patients undergoing chronic hemodialysis with a TCC. Case reports with fewer than five patients, non-English publications, and conference abstracts without full text were excluded. Priority was given to guideline statements such as KDOQI, KDIGO, CDC/HICPAC, IDSA, and CIRSE, as well as high-quality systematic reviews. Although a PRISMA flow diagram was not generated (given the narrative scope of this article), the methodology followed a structured, reproducible approach to ensure comprehensive coverage and transparency.

## 2 Tunneled central venous catheters in hemodialysis

A functional and reliable vascular access is the lifeline for patients undergoing chronic hemodialysis. The three main options are AVFs, AVGs, and TCCs. AVFs remain the gold standard due to their superior long-term patency and lower rates of infection and thrombosis. However, up to 80% of patients at dialysis initiation start hemodialysis treatment with a temporary non-tunneled central venous catheter. This often reflects the urgency of initianting dialysis, delayed referral for vascular access surgery, or anatomical constraints.

### 2.1 Indications for tunneled catheter use

TCCs represent an essential component of the vascular access strategy in end-stage kidney disease (ESKD) patients, although they are not considered the first-line option according to the 2019 Kidney Disease Outcomes Quality Initiative (KDOQI) Guidelines. Current recommendations emphasize the concept of the patient-centered ESKD Life-Plan, in which access choice is tailored to life expectancy, comorbidities, vessel anatomy, and preservation of future options. According to the most recent KDOQI guidelines, TCCs are mainly indicated in patients with exhausted peripheral access or vessels unsuitable for the creation of an AVF or AVG. They are also recommended as a bridging option while waiting for the maturation of a fistula or graft. In individuals with severe comorbidities, such as advanced heart failure, or in those with high surgical risk, a TCC may be the only feasible option. Similarly, in palliative settings or in patients with limited life expectancy, TCCs provide a minimally invasive and immediately functional form of access. Finally, patient preference can also justify catheter use, provided that the choice follows a shared decision-making process within the Life-Plan framework, taking into account quality of life and treatment goals. The 2019 KDOQI update stresses that even when a catheter is necessary, it should be seen as part of a succession plan. In practice, this means that patients who start dialysis with a catheter should have a definitive vascular access strategy defined within 30 days, in order to reduce prolonged catheter dependence and its associated complications, including infection, thrombosis, and central venous stenosis (6).

### 2.2 Anatomical sites, technical considerations and device durability

The choice of vascular access site for TCC placement is fundamental for ensuring long-term patency, reducing complications, and preserving future vascular options, in accordance with the KDOQI 2019 ESKD Life-Plan (6). The right internal jugular vein (RIJV) remains the preferred site, as it offers a short and straight trajectory to the superior vena cava and right atrium, with lower risks of kinking, recirculation, or stenosis. When this route is unavailable due to thrombosis, stenosis, or previous catheterization, the left internal jugular vein (LIJV) represents an acceptable alternative, although its longer and more angulated course increases the likelihood of malposition and dysfunction (7). The subclavian vein, although technically feasible, is usually avoided because of its strong association with central venous stenosis, reported in up to half of cases, which may jeopardize future creation of AVF or AVG (8). Its use is generally limited to exceptional or salvage situations, preferably under interventional radiology supervision. The femoral vein, on the other hand, is considered a last-resort option, typically reserved for patients with bilateral central venous occlusion or limited life expectancy. Compared with jugular approaches, femoral catheters carry higher risks of infection, shorter patency, and reduced patient mobility (9). Table 1 summarizes the recommended catheter lengths for each insertion site, ensuring appropriate tip positioning at the cavo-atrial junction or mid-right atrium.

**Table 1:** Suggested length ranges of TCC for different venous access sites, allowing optimal tip positioning at the cavo-atrial junction or mid-right atrium.

Insertion site	Recommended catheter length (cm)
Right internal jugular	19–31
Left internal jugular	23–36
Right femoral	36–55
Left femoral	≈55

Values are adapted from KDOQI 2019 and represent typical recommendations for adults. They are intended as a practical reference for planning insertion, with the understanding that individual anatomy and imaging guidance should always dictate final positioning.

**Figure 1:**
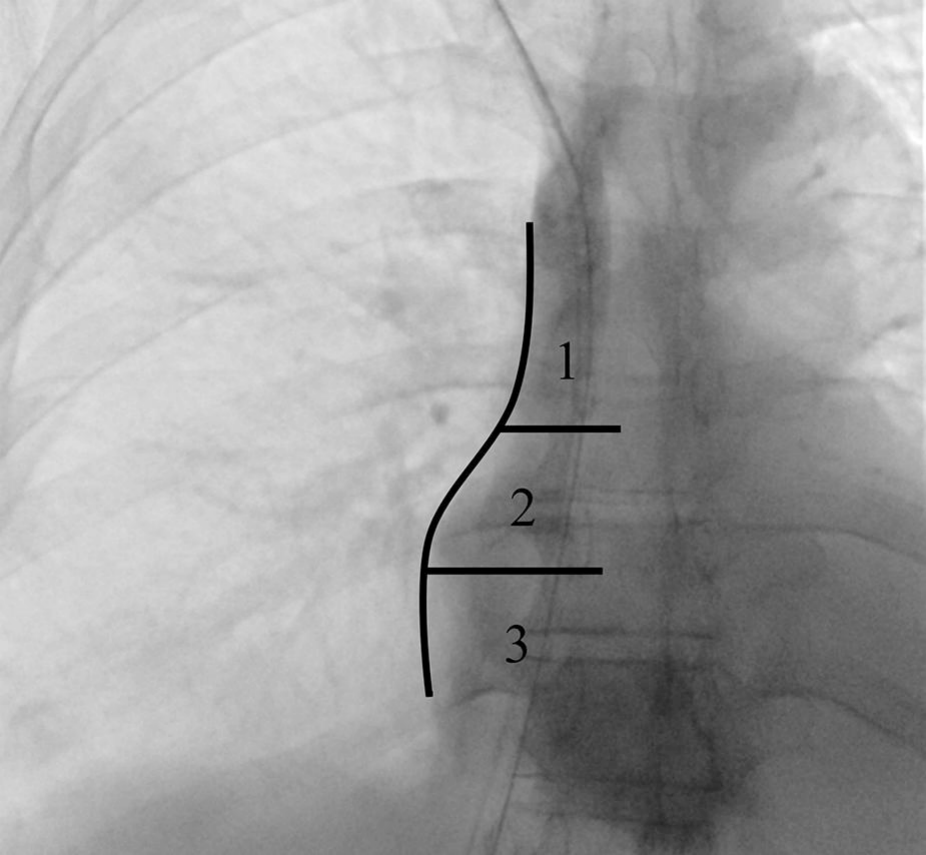
Fluoroscopic chest image in the supine position illustrating the three reference zones for central venous catheter tip assessment: (1) superior vena cava (SVC), (2) cavoatrial junction, and (3) mid- to deep right atrium. The concavity of the right atrial border (arrow) defines the transition between zones 1 and 2. Zone 2 extends for approximately one vertebral body height, with its superior and inferior margins aligned with the upper and lower endplates of consecutive vertebral bodies.

Technical accuracy is equally important. Real-time ultrasound (US) guidance during venipuncture is now regarded as the standard of care, as it markedly decreases arterial puncture, hematoma, and multiple unsuccessful attempts, all of which are more common with landmark-based access (10). Fluoroscopic imaging should be used to confirm that the catheter tip lies at the cavo-atrial junction or within the mid-right atrium (Figure 1), where flow is optimal and the risk of recirculation or dysfunction is minimized (11). The exit-site should be carefully chosen in an area away from skin folds, moist regions, or zones subject to repeated movement, as these factors increase the risk of infection or accidental dislodgment (12).

Modern catheters, usually made of polyurethane with step-tip or split-tip designs, are engineered to improve flow and reduce kinking. Mechanical problems, when they occur, typically involve external components such as clamps or hubs, which can often be repaired without catheter removal. Although several designs exist, no single device has shown clear clinical superiority, so choice should depend on institutional experience and patient characteristics (13, 14).

**Figure 2:**
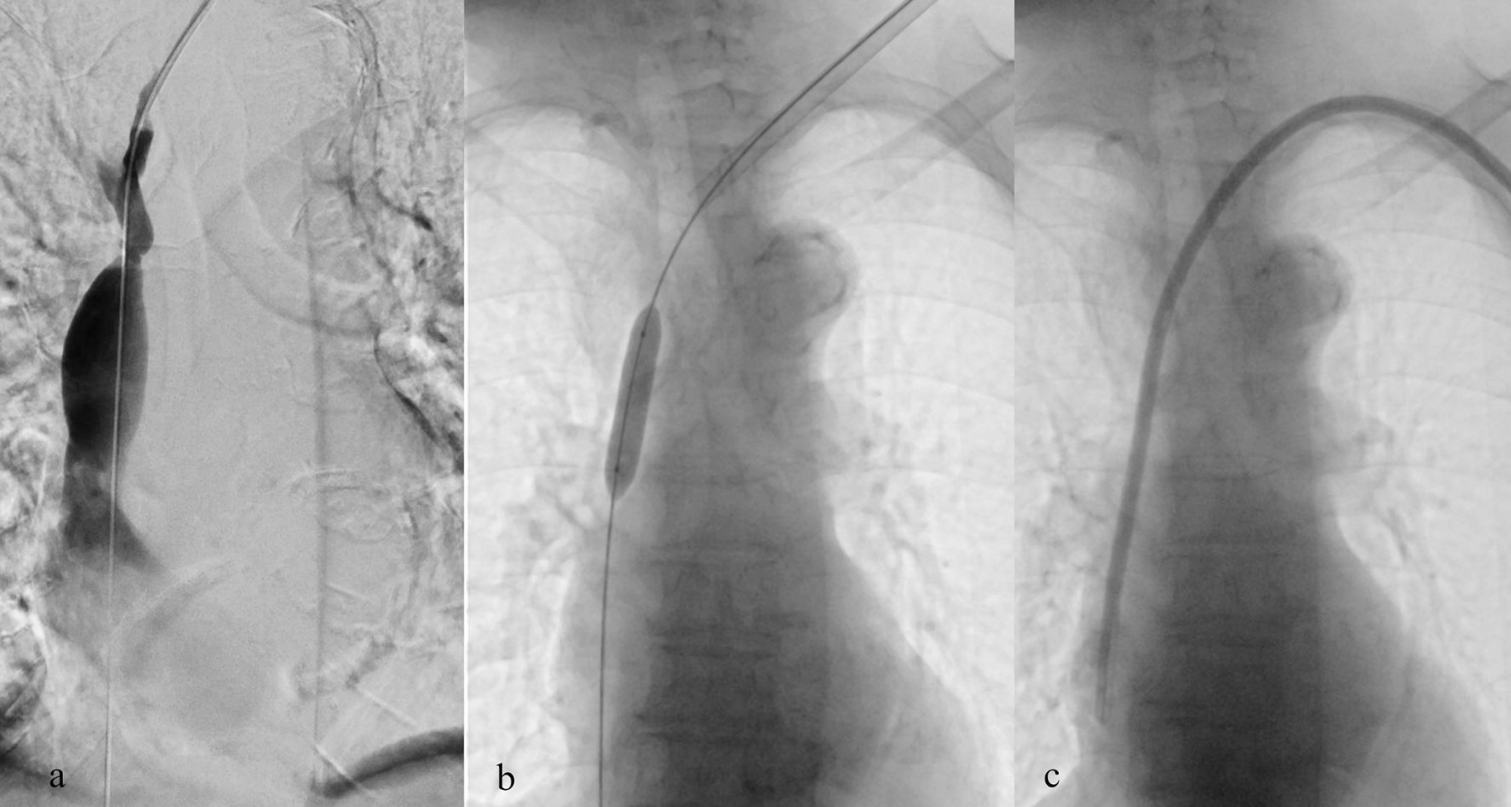
Placement of a TCC through the left internal jugular vein in a patient with significant stenosis of the left brachiocephalic vein **(a)**; balloon angioplasty performed at the stenotic tract **(b)** and successful placement of TCC **(c)**.

**Figure 3:**
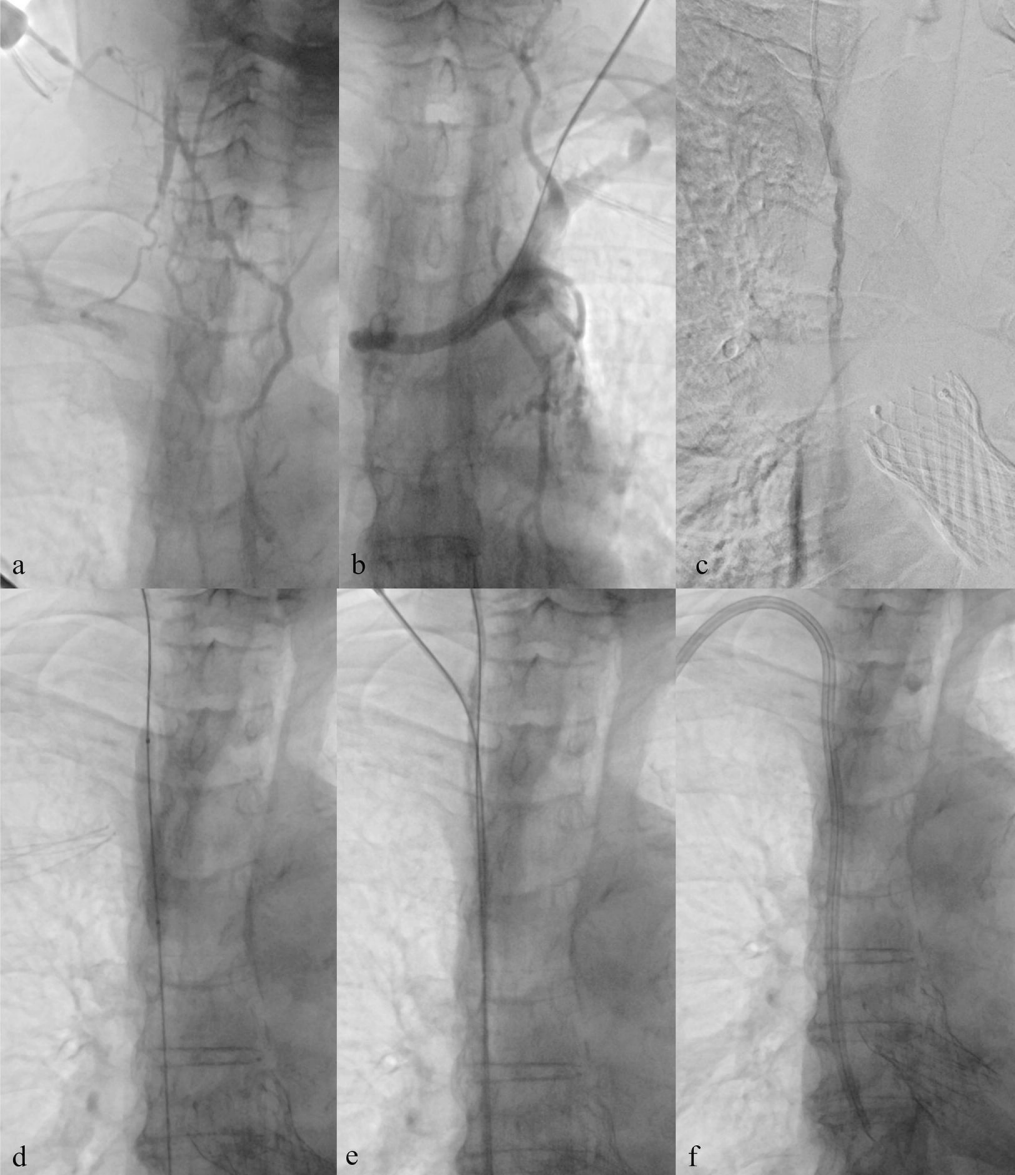
Central venous recanalization and tunneled catheter placement. **(a)** Diagnostic venography from a high puncture of the patent right internal jugular vein using an angiocath shows proximal occlusion of the right internal jugular vein with extensive collateral circulation, related to previous TCC. **(b)** Diagnostic venography through a 5 Fr introducer confirms occlusion of the left brachiocephalic vein, also due to prior TCC. **(c,d)** Recanalization of the occluded tract using a 0.035″ hydrophilic guidewire followed by balloon angioplasty. **(e,f)** Low puncture of the recanalized right internal jugular vein and successful placement of a new TCC.

In patients with prior catheterizations or venous stenosis, percutaneous transluminal angioplasty (PTA) can be employed to restore patency and allow catheter reinsertion at the same venous site. This approach avoids unnecessary use of alternative routes with higher complication risks (Figures 2, 3). Ultimately, site selection, procedural technique, and device choice must be considered together, always within a long-term strategy for vascular preservation consistent with the principles of the ESKD Life-Plan (6, 15).

### 2.3 Flow requirements for adequate hemodialysis

The main goal of any vascular access, AVF, AVG, or TCC, is to provide a stable blood flow that ensures effective dialysis. Insufficient flow compromises adequacy, leading to longer sessions, higher recirculation, and reduced solute clearance. Mature AVFs usually deliver flows of 600–1,200 mL/min, depending on vessel calibre, site and maturation. Persistent flows below 500 mL/min should raise suspicion for stenosis or thrombosis and often require prompt imaging and possible intervention (16). TCCs are expected to sustain extracorporeal flows of at least 300–400 mL/min to reach adequacy targets such as Kt/V ≥ 1.2 or URR ≥ 65%. Sustained reductions below these levels increase recirculation and compromise treatment (17). Routine monitoring of delivered blood flow during dialysis, supported by imaging when needed, allows early recognition of dysfunction and timely correction. This approach is consistent with vascular access quality initiatives and the long-term goals of the ESKD Life-Plan (6, 18).

## 3 Classification of complications

Complications related to TCCs for hemodialysis are differing in timing, severity, and clinical expression. They are often categorized as immediate, early, or late, a framework that helps guide both prevention and management. Immediate events occur during or soon after placement and usually reflect technical aspects or anatomical difficulties. Early complications develop within the first days to weeks, commonly linked to malposition, local inflammation, or malfunction. Late complications appear after prolonged use, typically as a result of biofilm formation, chronic infection, or progressive venous injuries (19, 20).

### 3.1 Immediate complications

**Figure 4:**
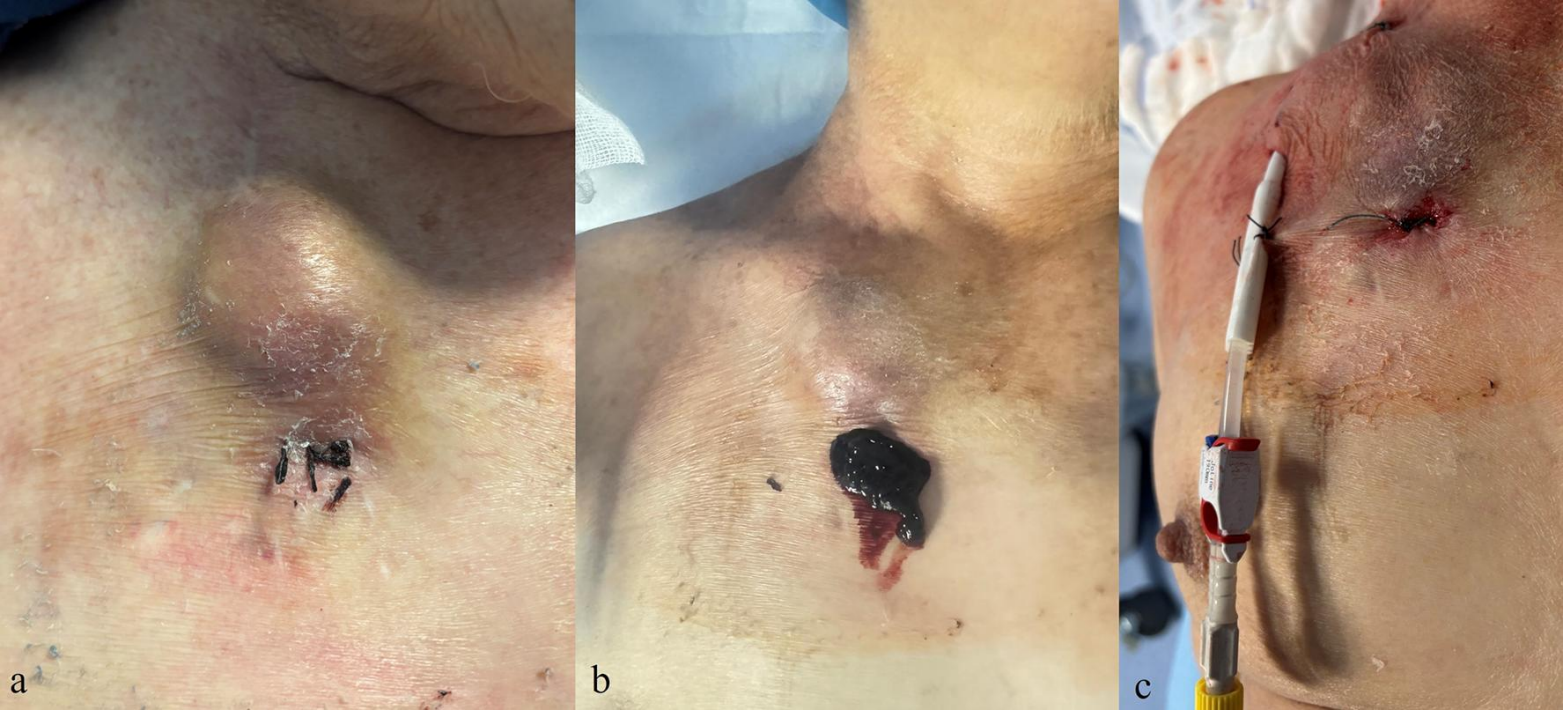
Clinical images of a complication after removal of a tunneled central venous catheter. **(a,b)** Hematoma at the exit-site with subsequent surgical evacuation. **(c)** Placement of a new tunneled central venous catheter at a different site.

**Figure 5:**
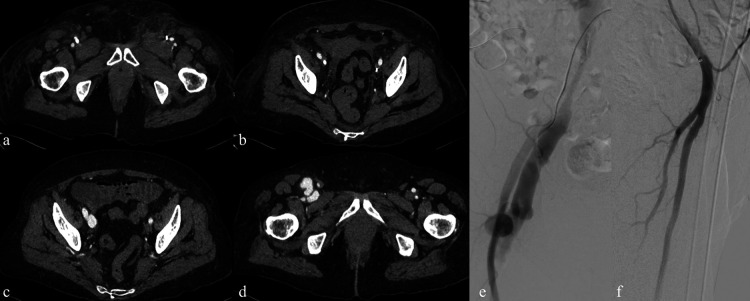
Imaging findings of a vascular complication related to temporary femoral central venous catheter placement for dialysis. **(a,b)** Contrast-enhanced CT showing a temporary dialysis catheter correctly positioned within the iliac vein but with inadvertent passage into the superficial femoral artery (circle). **(c,d)** Follow-up CT after catheter removal demonstrating the development of a pseudoaneurysm (arrow) and an arteriovenous fistula. **(e,f)** Digital subtraction angiography confirming the vascular injury and subsequent treatment with covered stent placement.

**Figure 6:**
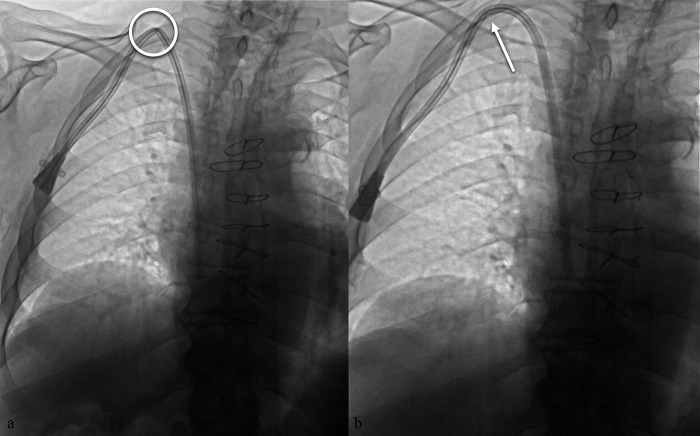
**(a)** malposition of a TCC with evidence of kinking along the subcutaneous course (circle). **(b)** Attempted repositioning by gentle device retraction, with persistence of a mild indentation (arrow).

**Figure 7:**
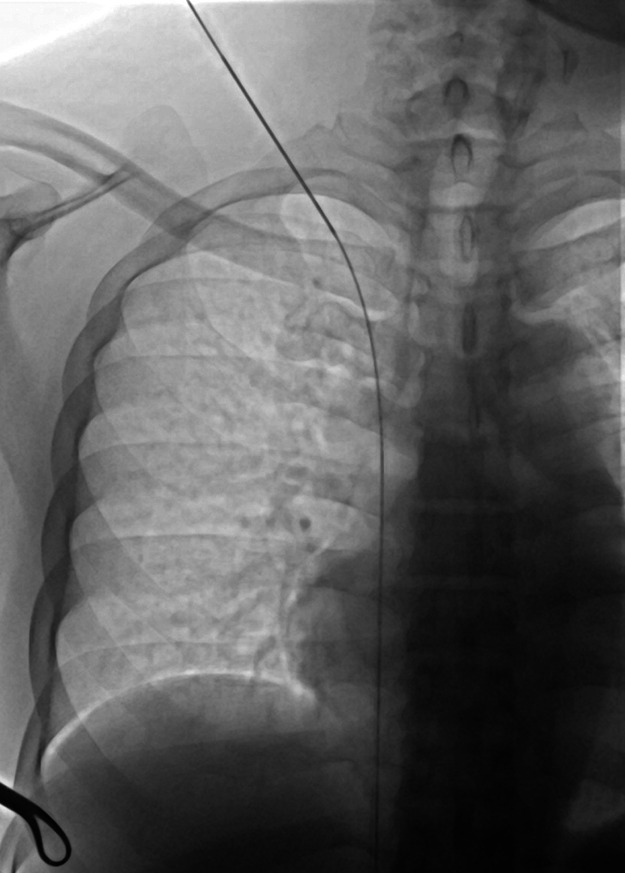
Fluoroscopic chest image in the supine position showing a low puncture site at the superior margin of the clavicle, with subsequent advancement of the guidewire from the superior vena cava directly into the inferior vena cava. The image highlights the anatomic relationship between the puncture site and the clavicle, an important landmark for safe and accurate venous access.

Despite advances in technique and routine use of US and fluoroscopy, acute events can still occur during placement (21). **Pneumothorax** (PNX), historically seen in 1%–6% of subclavian catheterizations, has become uncommon (<1%) with jugular access and US guidance (22, 23). **Hematomas** are reported in about 4% of US-guided punctures, rising to nearly 10% with landmark techniques, and are more frequent in anticoagulated patients or after multiple attempts (Figure 4) (24, 25). **Accidental arterial puncture** remains possible, particularly involving the carotid artery, with landmark approaches reporting rates of 4%–9% compared with <1% under US guidance (Figure 5) (22, 26). **Malposition** occurs in 1%–5% of procedures, despite fluoroscopy, and can lead to poor flow, thrombosis, or arrhythmias (Figures 6, 7) (7, 11). **Transient guidewire-induced arrhythmias** are also common, affecting up to one-third of insertions, but are usually benign and self-limiting (6).

### 3.2 Early complications

**Figure 8:**
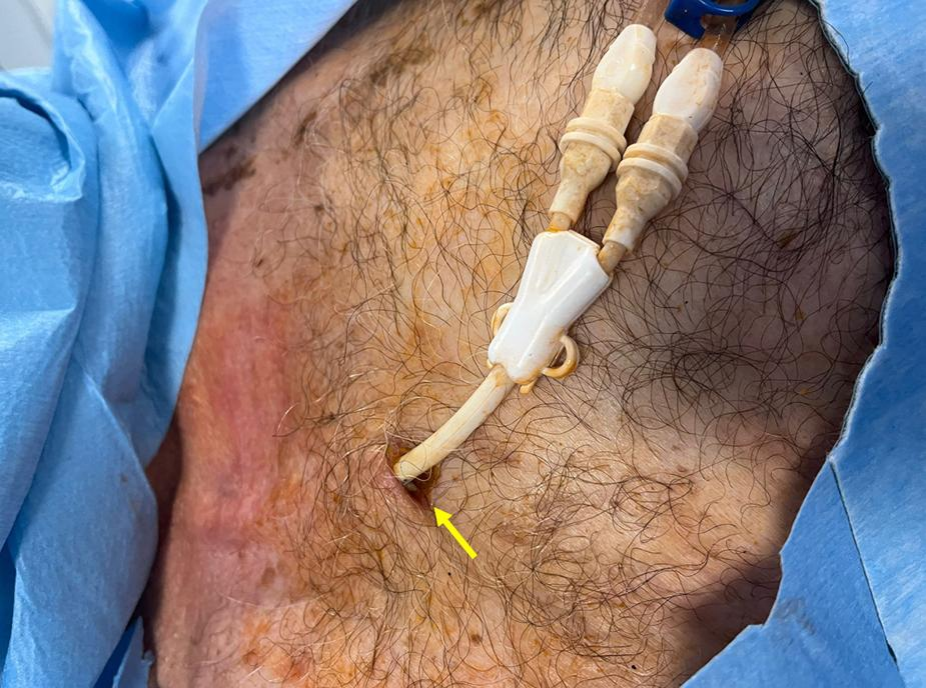
Exit-site infection of a tunneled cuffed catheter (TCC). Clinical image showing erythema, induration, and purulent discharge at the exit-site (yellow arrow). Exit-site infections are among the most common early complications of TCCs, and if not promptly recognized and treated, they may progress to tunnel infection or CRBSI.

**Figure 9:**
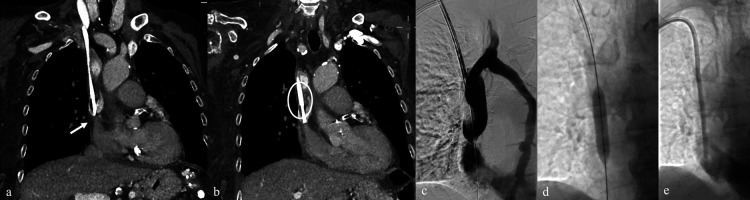
**(a,b)** Pre-removal CT of a TCC showing stenosis at the junction of the SVC and AD (arrow), associated with circumferential thrombotic deposition (circle). **(c)** Diagnostic venography performed via a 7 Fr introducer after TCC removal confirming CT findings, with collateral drainage through the hemiazygos vein. **(d,e)** Balloon angioplasty of the stenotic tract followed by successful placement of a new TCC.

These complications usually occur within the first two to four weeks after insertion. Timely recognition is essential to prevent progression to severe outcomes (22). **Exit-site infections**, reported at 0.5–1.6 per 1,000 catheter-days, are the most frequent, typically presenting with erythema, tenderness, and sometimes purulent discharge (Figure 8) (4, 27). **Early catheter dysfunction** is seen in 10%–20% of cases, often linked to malposition, kinking, or fibrin sheath formation, and usually manifests as reduced dialysis efficiency or high venous pressures (2, 11). **Intraluminal thrombosis** can affect 15%–20% of catheters within three months, particularly in the absence of standardized locking protocols (Figure 9) (28, 29).

### 3.3 Late complications

Long-term catheter use is associated with higher rates of infection and venous complications**. Catheter-related bloodstream infections (CRBSIs)** remain a leading problem, with rates of 2–5 per 1,000 catheter-days and cumulative risks of 25%–40% at one year (30–32). **Central venous thrombosis** develops in 10%–20% of long-term carriers and may be silent or present with limb or facial swelling, venous congestion, or loss of catheter function (9, 16, 33). **Central venous stenosis** occurs in about 20%–40% of patients and in more than half of those with prior subclavian catheterizations; symptoms include limb or neck swelling, collateral circulation, or impaired maturation of arteriovenous fistulas (15, 34). Finally, **biofilm formation** is almost universal in catheters left in place for more than one year, reported in over 90% of explanted devices; however, only a minor part of colonized catheters, about 20%–30%, evolve into overt infection, but biofilm is an important reservoir for recurrent bacteremia, dysfunction, and encrustation (17, 21).

## 4 Risk factors and predictors of complications

Recognition of the factors that predispose to TCC-related complications is fundamental to both prevention and clinical decision-making. These predictors can be grouped into patient characteristics, vascular anatomy, procedural aspects, and device-related features (35).

### 4.1 Patient-related factors

Advanced age, multiple comorbidities such as diabetes, cardiovascular disease, or malnutrition, and conditions of immunosuppression are all associated with impaired healing and increased susceptibility to infection. Patients on anticoagulants or with underlying coagulopathies are more prone to bleeding or hematoma. Body habitus may make venous access more technically challenging in obese patients, while cachexia and fragile skin may predispose to exit-site breakdown. Finally, a history of repeated catheterizations or prior central vein stenosis reduces the number of available access sites and raises the risk of mechanical complications (36, 37).

### 4.2 Procedural and operator dependent factors

Outcomes are closely tied to technique and operator expertise. Real-time US guidance reduces the risks of arterial puncture, hematoma, and failed attempts, while fluoroscopy ensures that the tip is correctly positioned at the cavo-atrial junction. Multiple blind punctures increase the likelihood of vascular injury, and errors in tunnelling may cause discomfort, kinking, or a higher risk of tunnel infection (38, 39).

### 4.3 Device and technique-related factors

Characteristics of the catheter itself also influence complication rates. Polyurethane devices, antimicrobial coatings, and lumen design may affect the risks of malfunction or infection. Longer dwell times inevitably increase the likelihood of infection and central venous pathology, underscoring the need for a long-term access plan. In addition, variability in the use of locking solutions and exit-site dressing protocols between centres contributes to differences in both infection and thrombosis incidence (15, 40).

## 5 Prevention strategies

Preventing complications in patient with TCC requires a comprehensive approach that starts before catheter placement and continues throughout the life of the device. This strategy combines accurate patient assessment, meticulous insertion technique, ongoing catheter maintenance, and education for both staff and patients. When implemented systematically, these measures can significantly reduce the risks of complications improving patient outcomes and preserving vascular access (41).

A careful pre-procedural planning should include a review of the vascular history, assessment of central vein patency with duplex US, and, in selected cases, cross-sectional imaging (CT or MR venography) in patients with multiple prior catheters or suspected central venous stenosis, to delineate venous anatomy and collateral pathways (42, 43). Attention to coagulation status and the temporary adjustment of antiplatelet/antithrombotic therapy is recommended in line with international protocols, as bleeding complications may occur even with meticulous technique. According to CIRSE quality improvement guidelines, coagulation status must always be checked and corrected, when necessary, since bleeding complications can occur even with careful technique. Minimum recommended thresholds include platelet count ≥50 × 10^9^ /L, INR ≤ 1.5, and aPTT ≤ 1.5 times control. When these values are exceeded, appropriate correction (platelet transfusion, plasma, or reversal therapy) should be undertaken prior to catheter placement. Whenever possible, elective TCC insertion should be scheduled after temporary suspension of these agents, balancing thrombotic and bleeding risks according to international peri-procedural management protocols (44).

Once the decision to proceed has been made, outcomes depend largely on operator expertise and adherence to aseptic standards. Sterile barriers, chlorhexidine-based skin antisepsis, and US-guided venipuncture are now regarded as essential components of safe practice, reducing the incidence of arterial puncture, hematoma, and failed access. Fluoroscopic imaging during insertion ensures accurate tip placement at the cavo-atrial junction or mid-right atrium, thereby minimizing malposition, recirculation, and dysfunction. The choice of the subcutaneous tunnel and exit-site also has important implications. Positioning the exit on the anterior chest wall, away from folds or moist areas, and securing the cuff within the tunnel reduces the risk of infection and accidental dislodgment, while post-procedural dressings help maintain a clean and dry environment (1, 45).

After placement, catheter preservation relies on consistent care and lock protocols. Heparin remains the most commonly used locking solution, while 4% citrate represents an accepted alternative with both anticoagulant and mild antimicrobial properties. However, current evidence does not demonstrate a clear superiority of citrate over heparin in preventing thrombosis or CRBI (46, 47). Periodic instillation of low-dose fibrinolytic locks, such as recombinant tissue plasminogen activator (rt-PA), has also been investigated as a preventive measure. In a multicenter randomized controlled trial, Hemmelgarn et al. reported that weekly rt-PA locks significantly reduced catheter dysfunction and infection rates compared with standard heparin, without increasing bleeding risk (48).

Daily practice in the dialysis unit plays a crucial role in maintaining catheter function and preventing complications. Each connection and disconnection should be performed under strict aseptic conditions, with meticulous hub disinfection and careful handling of the external components. The exit-site must be inspected at every dialysis session for signs of infection, ensuring that any early diagnosis is promptly recognized and addressed. Continuous monitoring of extracorporeal blood flow during dialysis provides valuable early indicators of dysfunction, allowing for timely imaging or intervention before complete failure occurs. A structured, standardized maintenance protocol, integrated into routine dialysis practice, remains one of the most effective measures to preserve catheter patency and prolong device lifespan (6, 42, 49).

Finally, patient education and institutional programs represent the foundation of long-term prevention. Patients should be instructed on how to keep the exit-site dry, protect the catheter during daily activities, and recognize early signs of infection such as redness, swelling, fever, or chills. At the same time, dialysis facilities are encouraged to maintain surveillance of their complication rates, benchmark them against national standards, and implement continuous quality improvement programs. These initiatives, supported by guideline statements such as KDOQI, demonstrate that systematic prevention strategies can substantially reduce morbidity, prolong catheter function, and preserve vascular access over time (50, 51).

## 6 Management of complications

Despite optimal technique and prevention protocols, complications remain frequent in hemodialysis patients. Their management requires early recognition and a coordinated approach aimed at reducing morbidity, avoiding treatment interruptions, and preserving vascular access. The therapeutic plan should be adapted to the specific type and severity of the event, as well as to the patient's clinical condition (6).

### 6.1 Infections

Infection is the most common and clinically significant complication. **Exit-site infections** are generally localized to the skin where the catheter emerges, presenting with erythema, tenderness, and sometimes purulent discharge but without systemic symptoms. When these are identified early, they can often be managed with oral or intravenous antibiotics targeting *Staphylococcus aureus* and coagulase-negative staphylococci, such as first-generation cephalosporins or cloxacillin (51). In areas with high methicillin-resistant Staphylococcus aureus (MRSA) prevalence, vancomycin is an appropriate empiric choice. Adjunctive care with topical agents, particularly mupirocin ointment or chlorhexidine-impregnated dressings, can further reduce the bacterial load and recurrence risk (52). **Tunnel infections***,* by contrast, involve the subcutaneous tract of the catheter and typically present with tenderness, swelling, or erythema that extends away from the exit-site. These infections carry a higher risk of bacteremia and generally require systemic antibiotics together with catheter removal and reinsertion at a different site. Conservative management without removal is rarely successful and risks progression to bloodstream infection (53). **CRBSIs** remain a leading cause of hospitalization and death in the hemodialysis population. They may present with fever, chills, rigors during dialysis, or as unexplained sepsis. Blood cultures should be drawn from both catheter lumens and, whenever possible, from a peripheral vein before antibiotics are started. A differential time to positivity greater than two hours between catheter and peripheral cultures strongly supports the diagnosis. Empiric therapy should be initiated promptly, typically with vancomycin for MRSA coverage plus an agent active against Gram-negative bacilli such as ceftazidime or cefepime and then tailored to culture results (31). The decision to attempt catheter salvage or proceed with removal depends on the organism, the patient's stability, and whether complications are present. Salvage may be considered in stable patients with infections caused by less virulent organisms, such as coagulase-negative staphylococci, when there is no tunnel infection. In these cases, systemic therapy is combined with antibiotic lock solutions instilled after each dialysis session. Removal, however, is strongly advised for infections caused by S. aureus, Candida species, Pseudomonas aeruginosa, multidrug-resistant Gram-negatives, persistent bacteremia beyond 72 h, or associated tunnel infection (53, 54). A particularly severe infectious complication is catheter-related right atrial thrombus, often linked to S. aureus bacteremia and associated with high mortality. This condition should be suspected in persistent bacteremia despite adequate therapy, especially if the catheter tip lies in the right atrium. Diagnosis is confirmed with echocardiography, and management generally involves catheter removal, prolonged systemic antibiotics, and anticoagulation until thrombus resolution. Surgery is reserved for large or refractory thrombi but carries significant operative risk (55).

### 6.2 Mechanical complications

Mechanical problems can occur during insertion or later in the life of the catheter and are a common cause of inadequate dialysis delivery (41). **Catheter malfunction** often results from intraluminal thrombus, fibrin sheath formation, kinking, malposition, or central venous stenosis. The initial management is usually the instillation of a fibrinolytic agent, such as 2 mg of alteplase per lumen, with a dwell time of 30–120 min before aspiration. If this does not restore adequate flow, fluoroscopic imaging can be used to assess tip position and detect kinking or malposition. In such cases, repositioning or exchanging the catheter over a guidewire is often successful. Fibrin sheaths may be disrupted mechanically during such procedures (56, 57). **Arterial injury** remains one of the most feared immediate complications of TCC insertion because of its potential severity. The carotid artery is the vessel most often involved during internal jugular access, and inadvertent puncture can result in hematoma, pseudoaneurysm, or arteriovenous fistula formation. Two clinical situations should be distinguished, as their management differs substantially. In cases of arterial puncture without catheterization, the appropriate response is immediate withdrawal of the needle, followed by firm manual compression of the puncture site for at least 10–15 min (longer in anticoagulated patients). US should then be used to exclude the presence of hematoma, pseudoaneurysm, or arteriovenous fistula. When promptly recognized and treated conservatively, most of these events resolve without sequelae, although short-term observation is advisable. If arterial catheterization with dilator or catheter advancement occurs, the management is more complex. The device should be left in place to prevent uncontrolled bleeding, and urgent imaging, typically CTA or angiographic study, should be obtained to assess the injury. Depending on the vessel involved, endovascular approaches may be feasible, including balloon tamponade, placement of a covered stent, or use of vascular closure devices; smaller arteries may be treated with coil or plug embolization. For large-caliber vessels such as the carotid, subclavian, or common femoral arteries, or in the presence of hemodynamic instability, surgical repair is usually required. Post-procedural surveillance with duplex US or CTA is recommended in all cases to ensure vessel patency and detect delayed complications such as pseudoaneurysm or fistula formation (25, 58). **Thoracic complications** such as PNX and hemothorax are less common with routine use of US guidance for venous puncture but remain important considerations. A small, asymptomatic PNX can often be managed with observation and supplemental oxygen, while larger or symptomatic collections require chest tube placement. When fluoroscopy is not used, obtaining a post-insertion chest radiograph is essential to detect these complications before they become clinically significant (45, 59). **Central venous stenosis** is frequently a late complication, often related to repeated use of the same central vein for access. Patients may present with swelling of the ipsilateral arm, neck, or face, and prominent collateral veins on the chest wall. The diagnosis is confirmed with venography or contrast-enhanced CT. PTA remains the first-line treatment, but restenosis rates are high, with reported recurrence in up to 50%–70% of patients within 6–12 months (60). Stent placement is recommended for elastic recoil, rapid restenosis, or recurrent lesions, as it offers better long-term patency than angioplasty alone. In particular, covered stents have demonstrated improved outcomes over bare-metal stents in maintaining vessel patency (34).

### 6.3 Thrombotic complications

Thrombotic events can affect the catheter lumen or the central veins and are a major contributor to catheter dysfunction. **Intraluminal thrombosis** is most often the result of blood reflux or inadequate flushing and presents with difficulty aspirating blood or reduced dialysis flow rates and which may be due to a fibrin sheath formation (57). Management involves fibrinolytic lock therapy, such as alteplase or urokinase, instilled into the lumen and allowed to dwell before aspiration (6). In patients with recurrent thrombosis, evaluation for an underlying hypercoagulable state should be considered; in selected cases, prophylactic use of anticoagulant lock solutions may help reduce recurrence (61). **Central venous thrombosis** presents with swelling of the ipsilateral limb, neck, or face, and sometimes with dilated collateral veins over the chest wall (33, 62). Management typically consists of systemic anticoagulation for a minimum of three months, using low molecular weight heparin or direct oral anticoagulants when appropriate; warfarin is less frequently used in the hemodialysis population due to bleeding risks (6). In severe or refractory cases, endovascular procedures such as catheter-directed thrombolysis or pharmaco-mechanical thrombectomy may be indicated (63, 64). Some thrombotic complications occur in association with infection: these require a combined therapeutic approach, including antimicrobial therapy, anticoagulation, and, in most cases, catheter removal to achieve definitive resolution (65).

The Table 2 summarizes the principal complications related to TCC and the recommended approaches for their management.

**Table 2:** Summary of the most relevant TCC complications, with corresponding first-line management, escalation strategies, and indications for catheter removal.

Complication	First-line approach	When to remove catheter	Adjunct/advanced options
Exit-site infection	Oral/IV antibiotics, topical antimicrobials	If tunnel infection develops	N/A
Tunnel infection	IV antibiotics	Usually required	Replace at new site
CRBSI	Systemic + lock antibiotics	S. aureus, fungi, severe sepsis, persistent bacteremia	Salvage in selected cases
Malfunction	Fibrinolytic instillation	If non-recoverable	Exchange/reposition
Arterial injury	Compression if needle only	If dilated and injured	Surgical repair
Pneumothorax	Observation if small	N/A	Chest tube if large
Intraluminal thrombosis	Fibrinolytic lock	If persistent	Evaluate hypercoagulability
Central venous thrombosis	Anticoagulation	N/A	Thrombectomy, thrombolysis
Central vein stenosis	PTA	N/A	Stent, bypass

Complications were selected based on their frequency and clinical relevance as reported in major guidelines (KDOQI 2019, IDSA 2009, CDC/HICPAC 2011) and in large contemporary series. The table is designed to provide an educational overview for clinicians, offering a concise, stepwise guide to prevention and treatment aligned with current evidence.

## 7 Current guidelines and evidence gaps

Several international documents provide guidance on the use of TCCs for hemodialysis. The most relevant are the KDOQI 2019 vascular access update (6), the CDC/HICPAC 2011 guidelines on the prevention of intravascular catheter infections (54), the IDSA 2009 guidelines on CRBSI (30), and the KDIGO 2024 guidelines for chronic kidney disease (CKD) (66). Together, they outline the main principles of insertion, maintenance, and infection control. However, there are differences in scope, strength of evidence, and level of detail. It should also be noted that the CDC and IDSA documents are more than a decade old and predate several technological and microbiological developments.

### 7.1 Current guidelines—key recommendations

**KDOQI 2019** guidelines are the most specific for vascular access. AVFs and AVGs should be prioritized as vascular access over catheters whenever possible; however, it recognizes that TCC remains essential for patients requiring urgent initiation of dialysis, those awaiting maturation of permanent access, and individuals with limited vascular options (see paragraph 2.1 Indications for Tunneled Catheter Use). For catheter placement, the RIJV is strongly preferred due to its favorable anatomy and lower risk of complications. Real-time US guidance is recommended for venipuncture, with fluoroscopic confirmation of tip placement at the cavo-atrial junction. Strict aseptic technique is mandatory, and procedures should be performed by trained, experienced operators. KDOQI also advocates minimizing catheter dwell time and initiating a plan for permanent access as soon as feasible, encouraging early planning for permanent access within 30 days from dialysis initiation (6). **CDC/HICPAC 2011** guidelines focus primarily on infection prevention for all central venous catheters. They recommend maximal sterile barrier precautions during insertion, including cap, mask, sterile gown, sterile gloves, and a full-body drape. Skin antisepsis with >0.5% chlorhexidine in alcohol is preferred, with tincture of iodine or 70% alcohol as acceptable alternatives when chlorhexidine is contraindicated. Daily review of the necessity of the catheter, strict adherence to hand hygiene, and standardized maintenance protocols are emphasized. The CDC also supports staff and patient education programs to improve adherence to infection prevention measures (54). **IDSA 2009** guidelines provide detailed recommendations for diagnosing and managing CRBSIs, representing the main reference. They define diagnostic criteria based on paired blood cultures and differential time to positivity, specify when catheter removal is indicated vs. when salvage may be attempted, and outline empiric antibiotic regimens. For patients with stable conditions and infections caused by less virulent organisms (e.g., coagulase-negative staphylococci), catheter salvage with systemic antibiotics and antibiotic lock therapy may be attempted. However, removal is advised for S. aureus, Candida species, multidrug-resistant Gram-negatives, and persistent bacteremia despite appropriate therapy. The IDSA also addresses tunnel infections, noting that these generally require catheter removal and systemic antibiotics. Although well-structured, these guidelines are outdated and do not reflect current resistance patterns (30). **The Kidney Disease: Improving Global Outcomes (KDIGO) 2024** guidelines align with KDOQI in promoting permanent access over catheters and highlight the need for early referral and multidisciplinary planning. They reinforce the use of US-guided cannulation, infection prevention bundles, and staff training. KDIGO further underscores the importance of tailoring vascular access choice to individual patient factors, including life expectancy, comorbidities, and personal preferences (66).

### 7.2 Comparative appraisal of major guidelines (KDOQI, KDIGO, CDC/HICPAC, IDSA)

Although the major guidelines broadly agree on the need to minimize catheter dependence and to prioritize permanent vascular access, their scope and level of detail differ substantially, which has important implications for clinical practice. All four guidelines agree on the main preventive measures: use of US for venipuncture, fluoroscopic tip confirmation, maximal sterile barrier protection, chlorhexidine-based antisepsis, and avoidance of routine catheter exchanges. They also support minimizing catheter dwell time and prioritizing early planning for permanent access. However, they differ in focus and depth. KDOQI provides the most practical recommendations for vascular access management. KDIGO gives strategic guidance on patient selection and care planning but does not address technical details. CDC guidance is prevention-oriented, while IDSA offers the only structured therapeutic approach for infection management. KDOQI is the only document to introduce the “30-day rule” for establishing a permanent access plan, while CDC and KDIGO recommend early planning without a specific timeframe. In the treatment of CRBSI, IDSA is the only guideline that clearly defines when to remove or attempt to salvage a catheter. The CDC guideline remains more general and prevention-focused. On the use of locking solutions, IDSA allows antibiotic locks for treatment in selected cases, whereas CDC discourages their routine use due to antimicrobial resistance concerns. KDOQI and KDIGO acknowledge their role but leave the choice to institutional protocols. In terms of evidence grading, there is substantial heterogeneity among the four major guidelines. The KDOQI 2019 update applies the GRADE framework, with most recommendations for TCCs supported by moderate- to low-quality evidence (Grade B–C) and a limited number rated as high-quality (Grade A), mainly regarding aseptic technique and ultrasound guidance. The KDIGO 2024 guideline also uses GRADE but integrates vascular access within broader CKD care, citing evidence mostly of moderate or low certainty (2B–2C). The CDC/HICPAC 2011 document employs its own classification system (Category IA–II) rather than GRADE: key measures such as maximal sterile barriers and chlorhexidine antisepsis are Category IA, reflecting strong evidence or regulatory consensus, while recommendations specific to dialysis catheters remain Category II, based on expert opinion. The IDSA 2009 guideline, using the A–E/I–III hierarchy, includes several A-II or A-III recommendations for CRBSI management and only a few A-I statements supported by randomized trials. Notably, less than 10% of all recommendations in the reviewed guidelines are supported by high-quality (A-I or 1A) evidence, highlighting the predominance of consensus-based statements. From a practical standpoint, the inconsistent quality of evidence across all major guidelines directly contributes to variability in how TCC is implemented worldwide. While KDOQI provides detailed procedural guidance, its recommendations are frequently based on moderate- or low-certainty evidence, reflecting the scarcity of controlled studies. KDIGO, conversely, adopts a strategic, policy-level perspective that lacks sufficient procedural depth, creating a disconnection between planning and bedside execution. The resulting gap means that decisions on catheter placement, maintenance, and salvage still depend heavily on local expertise rather than uniform standards. In contrast, the CDC and IDSA guidelines, though historically influential, are now outdated both in methodology and in clinical relevance. Their evidence base predates the widespread emergence of multidrug-resistant organisms, the use of biofilm-active agents such as taurolidine or ethanol locks, and the integration of imaging-guided endovascular approaches into routine practice. This temporal lag limits their applicability to current hemodialysis populations and may even perpetuate outdated infection-control practices. Collectively, these discrepancies underscore the fragmented nature of existing guidance: KDOQI defines how procedures should be performed, KDIGO defines why and when, while CDC and IDSA define how to prevent or treat complications, but none provide a cohesive, evidence-graded framework that integrates these dimensions. A unified update, dialysis-specific and multidisciplinary in scope and evidence-based, is therefore urgently needed to bridge the gap between evidence, policy, and clinical reality (6, 30, 56, 66).

### 7.3 Evidence gaps and future needs

Despite the comprehensive nature of these guidelines, several critical gaps in evidence and practice remain. One notable area is the absence of high-quality comparative studies evaluating different lock solutions; while heparin remains the most widely used, emerging alternatives, such as taurolidine, ethanol, citrate, and antimicrobial peptide-based locks, show promise in reducing CRBSIs, but direct head-to-head trials across diverse patient populations are scarce. As a result, practice varies widely between institutions (47, 67, 68).

Another gap concerns the optimal dwell time for TCCs and whether there should be a threshold for elective replacement in asymptomatic patients. There is currently no universal consensus on this issue, and decisions are often driven by local policy or individual clinician judgment (69, 70).

Exit-site care protocols also vary considerably. Differences in dressing type (transparent adhesive vs. gauze), frequency of changes, and choice of antiseptic agent are common, yet comparative evidence is limited. While chlorhexidine-impregnated dressings have shown benefit in reducing infection risk in some studies, cost, skin sensitivity, and patient comfort remain factors influencing choice (3, 49, 71).

Definitions and reporting standards for catheter-related complications, particularly non-infectious events such as central vein stenosis or fibrin sheath formation, are inconsistent across studies and registries. This lack of uniformity makes it difficult to compare outcomes, assess the true burden of complications, or measure the impact of preventive strategies (45, 52).

Operator-related factors are another underexplored area. Although observational data suggest that procedures performed by high-volume, image-guided operators, such as interventional radiologists, are associated with better outcomes, there is limited prospective research comparing complication rates between specialties, such as nephrology, surgery, and radiology (69, 72).

Future research should focus on multicentre trials using standardized definitions and outcome measures. In addition, the integration of advanced imaging tools such as intravascular US (IVUS) may enhance the understanding and management of venous pathology, particularly in patients with catheter-related thrombosis or central venous stenosis (73). Similarly, innovative techniques like US-only placement of TCC, recently reported as feasible and safe, could in time reduce reliance on fluoroscopy, though their broader validation and standardization are still needed (74, 75). Until stronger evidence emerges, a multidisciplinary, patient-centered approach remains the most practical and evidence-based strategy.

## 8 Conclusion

TCCs remain a necessary but temporary solution for patients who cannot yet receive an AVF or AVG. Despite advances in materials, imaging, and prevention, infection, thrombosis, and catheter dysfunction continue to cause substantial morbidity. The RIJV, US-guided venipuncture, and strict asepsis remain the safest procedural standards. Long-term success depends not only on technical skill but also on standardized maintenance, early planning for permanent access, and collaboration among nephrology, surgery, and interventional radiology. Existing guidelines provide a solid foundation but require updating to reflect current practice and emerging technologies. Incorporating new tools such as IVUS and US-only placement techniques may further improve safety and vessel preservation. The ultimate goal is to ensure that TCCs serve as a safe bridge, not a permanent solution, for patients requiring hemodialysis.
